# Case Report: Laboratory detection of a thrombotic tendency in a family with hypodysfibrinogenemia and a novel *FGG* mutation

**DOI:** 10.3389/fcvm.2024.1488602

**Published:** 2024-10-15

**Authors:** Amaury Monard, Elisabetta Castoldi, Ilaria De Simone, Kanin Wichapong, Tirsa van Duijl, Maartje van den Biggelaar, Stefano Spada, William van Doorn, Dave Hellenbrand, Paola van der Meijden, Frauke Swieringa, Alexander Stork, Hugo ten Cate, Erik Beckers, Floor Heubel-Moenen, Yvonne Henskens

**Affiliations:** ^1^ Department of Internal Medicine—Hematology, Maastricht University Medical Centre+, Maastricht, Netherlands; ^2^ CARIM, School for Cardiovascular Disease, Maastricht University, Maastricht, Netherlands; ^3^ Department of Biochemistry, CARIM, Maastricht University, Maastricht, Netherlands; ^4^ Department of Molecular Hematology, Sanquin Research, Amsterdam, Netherlands; ^5^ Centre for Thrombosis and Hemostasis (CTH), University Medical Centre Mainz, Mainz, Germany; ^6^ Central Diagnostic Laboratory, Maastricht University Medical Centre+, Maastricht, Netherlands; ^7^ Thrombosis Expertise Center, Heart+ Vascular Center, Maastricht University Medical Center+, Maastricht, Netherlands; ^8^ Department of Internal Medicine, Anna Hospital, Geldrop, Netherlands; ^9^ Department of Internal Medicine, Maastricht University, Maastricht, Netherlands

**Keywords:** congenital fibrinogen disorders, hypodysfibrinogenemia, diagnosis, phenotype, case report

## Abstract

**Introduction:**

Hypodysfibrinogenemia is a rare congenital fibrinogen disorder (CFD) which may induce thrombotic and bleeding events. Therefore, patient management needs careful evaluation. Routine coagulation tests are inadequate to predict the clinical phenotype.

**Clinical findings:**

A 60-year-old woman with both bleeding and thrombotic complications and her two daughters were referred to our center for genotypic and phenotypic analysis of a CFD.

**Diagnosis:**

Conventional laboratory results led to the diagnosis of hypodysfibrinogenemia in all three subjects. They all carried the same heterozygous c.1124A>G mutation in *FGG* resulting in p.Tyr375Cys amino acid substitution, which was confirmed by protein variant analysis from plasma. *In silico* structure analysis predicted possible conformational and functional changes of the fibrinogen molecule. Thrombin generation indicated a hypercoagulable state confirmed by microfluidics that showed enhanced fibrin formation in both daughters, regardless of the coagulation trigger.

**Conclusion:**

We report on a family with hypodysfibrinogenemia and a novel *FGG* heterozygous missense mutation, possibly leading to conformational changes or covalent dimerization. Thrombin generation and particularly microfluidic measurements disclosed a hypercoagulable state, which was not detected with routine coagulation tests, justifying a different patient management.

## 1 Introduction

Congenital fibrinogen disorders (CFDs) are rare and clinically heterogeneous haemostatic disorders that can be classified as quantitative (type I) or qualitative (type II) (1, 2). The associated clinical phenotype ranges from bleeding to thrombosis and cannot be easily predicted from routine laboratory test results, especially for qualitative defects (1–5). This makes the management of these patients complex. Follow-up is recommended even in asymptomatic patients, as they are prone to developing both thrombotic and bleeding events (3).

In the last years, global haemostatic assays such as the viscoelastic ROTEM test, thrombin generation and thrombus formation in the Maastricht Flow Chamber, have been developed to assess the overall hemostatic balance. These assays could provide information on the bleeding or thrombotic tendency in patients with CFDs (6–8). Here we present a family with congenital hypodysfibrinogenemia associated with a novel *FGG* gene mutation, and we report on the performance of global hemostasis assays in elucidating the clinical phenotype.

## 2 Case report

A 60-year-old woman, who was diagnosed with congenital hypofibrinogenemia in another center at the age of 26 years, was referred to our hemophilia treatment center (HTC) for a second opinion. Her medical history was remarkable for three spontaneous miscarriages. The patient underwent the first spontaneous abortion at 22 weeks gestation when she was 26 years old. This miscarriage was caused by a placental infarction, followed by a major fluxus during partus, for which a blood transfusion was required. Subsequent analysis for bleeding tendency uncovered low fibrinogen levels, but no other abnormalities predisposing to bleeding. A diagnosis of congenital hypofibrinogenemia was made at that time. Shortly after the first miscarriage, the patient presented with pulmonary embolism (PE).

Two additional spontaneous abortions occurred at 13 weeks gestation at the age of 27 years and 28 years, respectively. After these three consecutive miscarriages, during the next pregnancies she received prophylactic treatment with cryoprecipitate two times a week and therapeutic low-molecular weight heparin (LMWH). This resulted in two successful pregnancies, out of which two daughters were born. Both daughters were tested immediately after birth and were diagnosed with hypofibrinogenemia. No further bleeding or thrombotic events occurred in the proband until the age of 59 years, when she presented with a second episode of spontaneous PE. Anticoagulant therapy with dabigatran was started. After this second thrombotic event, the patient and her two daughters were referred to our HTC for genotypic and phenotypic evaluation, therapeutic advice in case of bleeding or surgical or dental interventions, and guidance for future pregnancy and delivery (daughters).

The daughters of the proband were 31 (daughter 1) and 23 (daughter 2) years old upon referral. Daughter 1 suffered from menorrhagia, which normalized after initiation of an oral contraceptive. She had not been challenged by surgical interventions, dental extractions, pregnancies, or deliveries yet. The International Society on Thrombosis and Haemostasis bleeding assessment tool (ISTH-BAT) score at the time of referral was 2. There was no history of thrombotic events. Daughter 2 had no history of bleeding tendency or thrombotic events and her ISTH-BAT score was 0. She also used an oral contraceptive at the time of referral. The ISTH-BAT score of the proband was 3.

**Figure 1:**
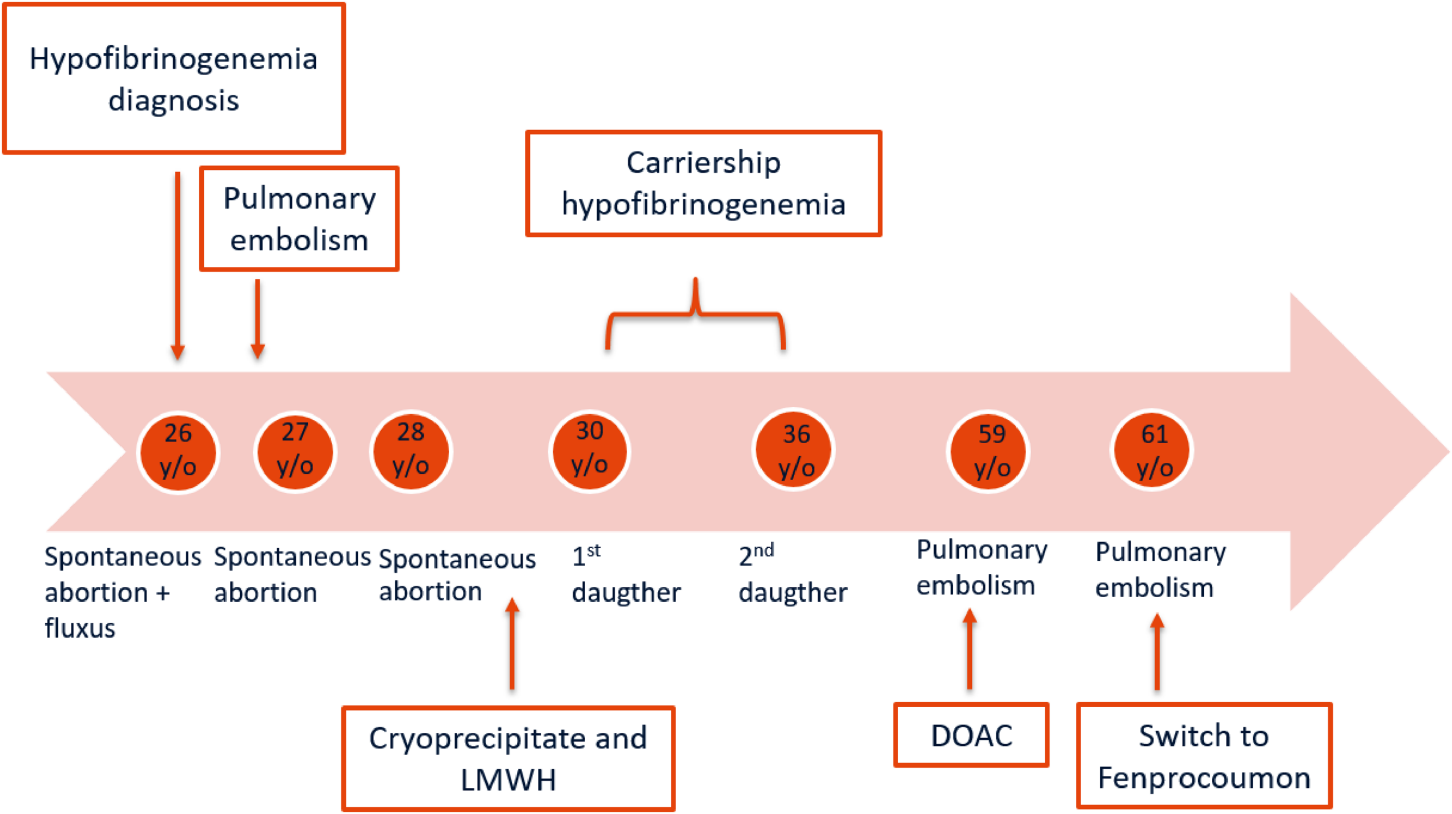
Timeline of the episodes of care of the proband.

Approximately one year after the proband was referred to our HTC, she presented with spontaneous PE (third episode) whilst being treated with dabigatran. About six months earlier, the level of dabigatran was 58 ng/mL, measured two hours after dabigatran intake. Taking into consideration the dosage and the time of administration, this level was just below the expected range of 60–450 ng/mL (9) which might have played a role in the PE recurrence. Anticoagulant treatment was switched to fenprocoumon combined with therapeutic LMWH as bridging therapy until the INR was adequate. The timeline of the proband's medical history is schematically presented in Figure 1.

## 3 Diagnostic assessment

### 3.1 Diagnostic testing

#### 3.1.1 Conventional laboratory testing

Conventional laboratory test results are shown in Table 1. The prothrombin time (PT) was prolonged and reptilase time was outside the reference range in all three subjects. In the proband, fibrinogen activity was decreased whilst fibrinogen antigen levels were within reference ranges. Differently, both fibrinogen antigen and activity levels were decreased in the daughters. Fibrinogen test results in all three subjects point in the direction of hypodysfibrinogenemia (fibrinogen activity/antigen ratio <0.7). They classify as mild hypodysfibrinogenemia (type 4C) as their fibrinogen antigen levels are between 1 g/L and the lower limit of the normal range (1). The higher fibrinogen antigen levels in the proband compared to her daughters might be explained by the age-dependent increase of fibrinogen antigen levels (10).

**Table 1:** Demographic and clinical characteristics, and results of clinical laboratory measurements in the proband and daughters.

Laboratory measurements	Normal range	Proband	Daughter 1	Daughter 2
Age (years)		60	31	23
Gender		Female	Female	Female
Medication		Dabigatran	Oral contraceptive	Oral contraceptive
Clinical features		Haemorrhagic and thrombotic events	Menorrhagia	Asymptomatic
Phenotype		Hypodysfibrinogenemia	Hypodysfibrinogenemia	Hypodysfibrinogenemia
ISTH-BAT score	0–6	3	2	0
Thrombin time (s)	15–22	^a^	18.9	**22.2**
Prothrombin time (s)	9.9–12.4	**13.4**	**14.6**	^b^
Reptilase time (s)	17–25.4	**26.9**	**27.9**	**28.6**
Fibrinogen antigen (g/L)	1.8–3.0	1.87	**1.25**	**1.04**
Fibrinogen activity (g/L)	1.7–4.0	**0.8**	**0.6**	**0.5**
Fibrinogen activity to antigen ratio		0.4	0.5	0.5
Factor XIII activity (%)	70–140	76	**55**	**51**
Plasminogen activity (%)	80–120	^c^	**137**	**131**
Genotype		Heterozygous	Heterozygous	Heterozygous
Gene		*FGG*	*FGG*	*FGG*
Nucleotide		c.1124A>G	c.1124A>G	c.1124A>G
Amino acid		p.Tyr375Cys	p.Tyr375Cys	p.Tyr375Cys

Results that are outside the reference ranges are marked in bold.

^a^ Not reliable due to dabigatran treatment.

^b^ Prothrombin time could not be measured due to very low levels of fibrinogen.

^c^ Not measured.

Plasminogen levels were slightly elevated in both daughters, whereas factor XIII (FXIII) activity levels were mildly decreased. This is probably a test artefact due to low fibrinogen levels, as described in the Siemens Healthineers Berichrom FXIII manual (11).

Factor V Leiden and Prothrombin G20210A were absent. Activated protein C (APC) resistance, protein C activity, total and free protein S antigen, and lupus anticoagulant were all within the normal range (data not shown). In the proband, thrombophilia workup was done under dabigatran as this treatment could not be discontinued. For these assays, DOAC-remove was used before carrying out the measurements.

#### 3.1.2 DNA sequence analysis

DNA sequence analysis of the *FGA* and *FGB* genes showed no abnormalities. Analysis of the *FGG* gene showed the presence of a novel heterozygous variant (c.1124A>G) predicting the p.Tyr375Cys amino acid substitution in all three subjects. This variant is located in a functional domain of the *FGG* gene in which multiple pathogenic missense variants have been reported (12). This variant was classified as likely pathogenic (class 4), based on the American College of Medical Genetics and Genomics (ACMG) variant classification criteria (13).

#### 3.1.3 Fibrinogen protein analysis

**Figure 2:**
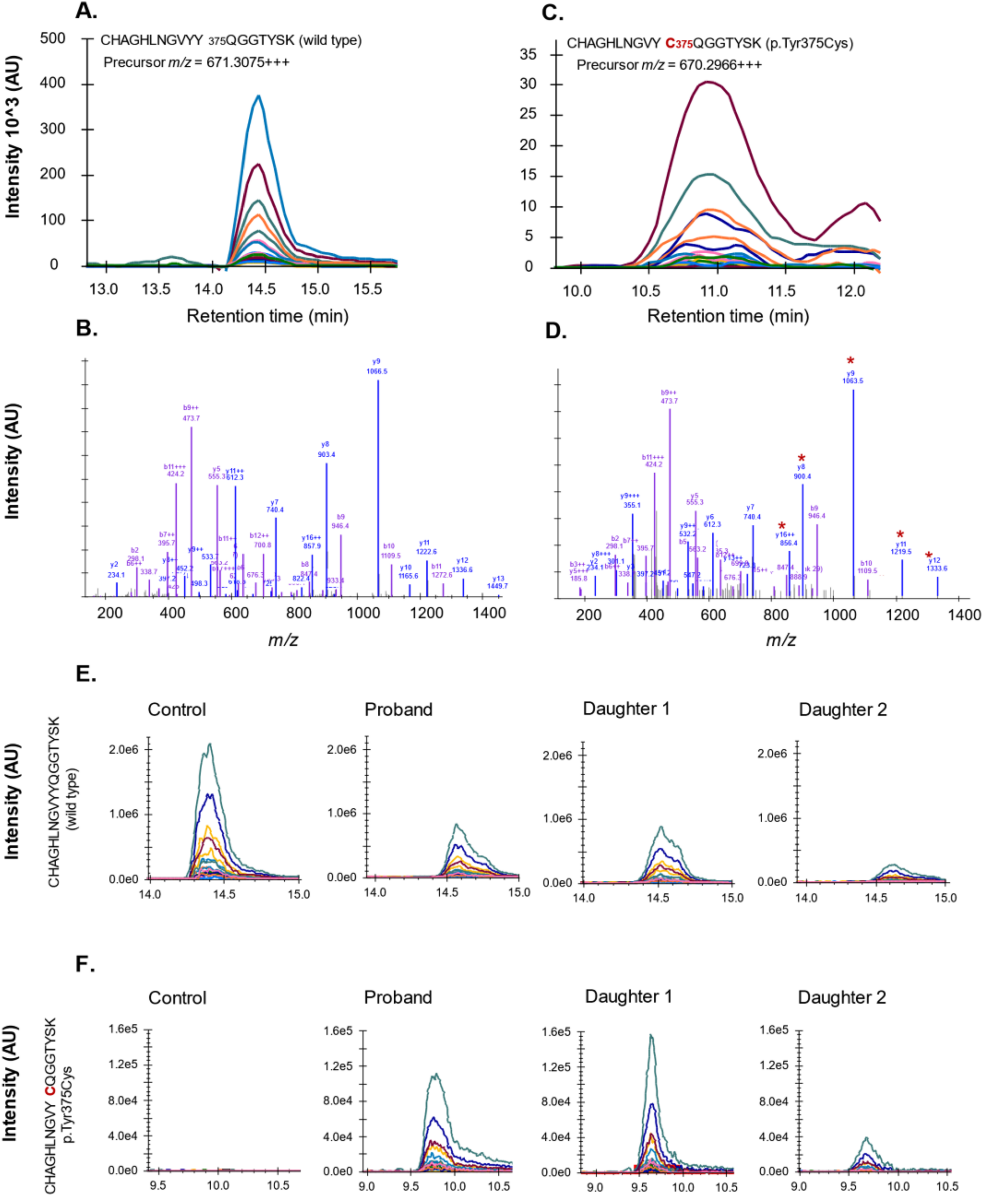
Confirmation of p.Tyr375Cys amino acid substitution by mass spectrometry analysis of fibrinogen γ chain from plasma. Peptides unique to wild-type **(A,B)** or variant **(C,D)** fibrinogen γ chain were quantified in plasma. LC chromatogram and mass spectra shown with product fragments unique to the variant sequence indicated by asterisk. **(E)** Tryptic peptide representing wild-type fibrinogen γ chain in pooled normal plasma (control) and plasma of studied family members. **(F)** Tryptic peptide representing variant p.Tyr375Cys measured in pooled normal plasma (control) and plasma of studied family members.

The presence of this missense mutation at the protein level was confirmed by mass spectrometry (LC-MS/MS) analysis of plasma fibrinogen (Figure 2). Both fibrinogen γ chain-derived peptide sequences, comprising a tyrosine (Tyr) or cysteine (Cys) at position 375, were identified in plasma from the proband and her daughters, whereas only the wild-type p.Tyr375 sequence was detected in control plasma.

The newly introduced cysteine residue is located near the A-hole, a functional site involved in fibrin polymerisation (14). As indicated by *in silico* analysis of the crystal structure of fibrinogen (PDB ID 3GHG) and molecular dynamics (MD) simulations, this free cysteine (p.Tyr375Cys corresponding to Tyr349 residue in the PDB structure) could form spurious disulfide bonds with cysteines 339 and 326 of the same γ chain, causing a significant conformational change of the A-hole that could affect fibrin polymerisation (Supplementary Figure S1). Alternatively, Cys375 could promote disulfide bonding between mutant γ chains, resulting in grossly abnormal fibrinogen molecules that are unlikely to be secreted. Accordingly, the 375Cys γ chain variant was strongly under-represented in plasma of the three mutation carriers (as assessed by mass spectrometry) and no structural abnormality could be detected by Western blot analysis of their plasma fibrinogen (data not shown). Taken together, these observations could account for the hypodysfibrinogenemia observed in carriers of the *FGG* p.Tyr375Cys mutation.

Approximately 10% of all fibrinogen molecules contain a splicing isoform of the γ chain, known as γ' chain, that mediates high-affinity binding of fibrinogen to thrombin and inhibits thrombin-catalyzed activation of platelets, FV and FVIII (15–17). Since the fibrinogen γ' fraction has been inversely associated with the risk of venous thrombosis (18), we also measured fibrinogen γ’ levels. ELISA assays showed that fibrinogen γ' levels in the mother (63.5% of pooled normal plasma), in daughter 1 (44.0%) and in daughter 2 (32.8%, measured after discontinuation of the pill) were decreased in proportion to their total fibrinogen levels, indicating that the identified *FGG* variant does not alter the ratio between the two fibrinogen γ chain isoforms.

#### 3.1.4 Thrombin generation

Thrombin generation [Calibrated Automated Thrombography (19) in platelet-poor plasma] could only be measured in the daughters, because the proband was on anticoagulant treatment. Both daughters were using oral contraceptives at the time of testing, but daughter 2 was also re-assessed after discontinuation of this medication. At low tissue factor (TF), both daughters showed slightly shorter lag times and higher peaks of thrombin generation than pooled normal plasma, indicating a mild hypercoagulable state. However, this disappeared when daughter 2 was re-tested off the pill. When thrombin generation was measured at high (10 pM) TF and soluble TM was added to probe the protein C pathway, both daughters showed mild APC resistance with residual endogenous thrombin potential (ETP) of 72% and 60% respectively, vs. 42% in pooled normal plasma. Interestingly, although the residual ETP in daughter 2 decreased to 51% after discontinuation of oral contraceptive use, it did not normalize completely (Supplementary Figure S2).

#### 3.1.5 Microfluidic assay

**Figure 3:**
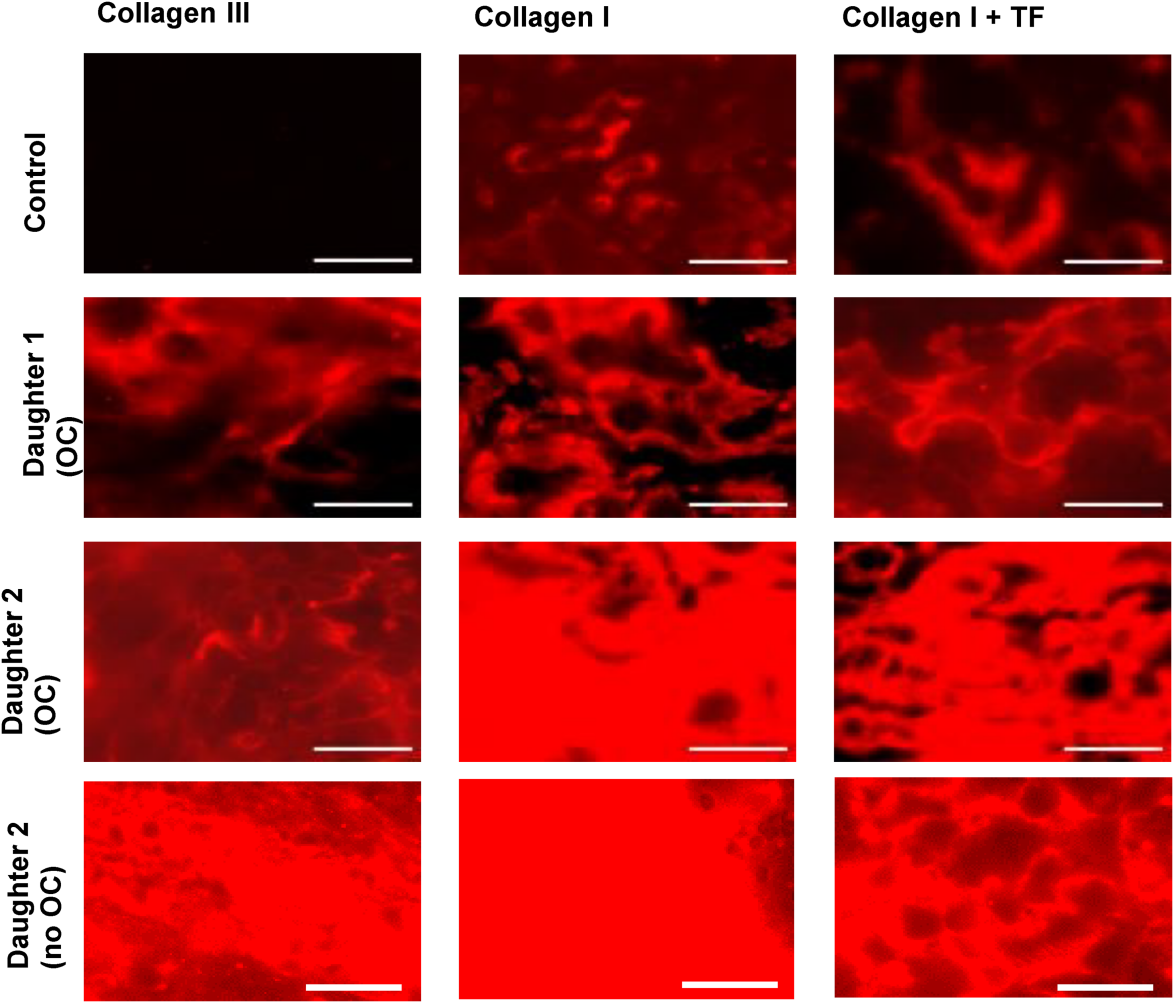
Fluorescence microscopic images from the microfluidic assay showing fibrin deposition, imaged at *t* = 10 min. Alexa Fluor-647 fibrinogen was added to whole blood to visualize fibrin formation. Citrate blood samples were recalcified with CaCl_2_/MgCl_2_ and perfused over collagen III or collagen I ± tissue factor (TF), at a shear rate of 1,000 s^−1^. Data obtained from daughters 1 and 2 were compared to 6 healthy donors (1 representative control is shown). Scale bar = 40 µm.

Both daughters were also tested in a microfluidic assay of thrombus formation under flow using the Maastricht Flow Chamber (Figure 3). In this assay, whole blood was perfused over surfaces coated with collagen type III or collagen type I with or without TF, and platelet deposition and fibrin formation were monitored using fluorescent probes (10). Platelet deposition on the collagen surfaces was similar between both daughters and controls, whereas the aggregate coverage on the relatively weak agonist collagen type III was remarkably higher in the daughters (Supplementary Figure S3). In control blood, fibrin was solely formed on the surface where collagen type I was co-coated with TF. In contrast, in the daughters' blood, fibrin formation was detected on all surfaces, regardless of TF triggering of coagulation (Figure 3). Additional data on surface area coverage (%SAC) over time can be found in Supplementary Figure S3. Although both daughters were using oral contraceptives at the time of testing, re-assessment of daughter 2 when she was no longer using oral contraceptives (after three months) yielded similar results to the first measurement, suggesting a minor contribution of oral contraceptive use to the procoagulant effects observed in this assay.

## 4 Therapeutic intervention and follow-up

Based on the extensive geno- and phenotyping, a personal treatment plan was set-up for all three subjects. For the proband, the treatment plan included advice concerning prophylaxis and treatment for both thrombotic and hemorrhagic risk situations. In case of bleeding or a surgical intervention, administration of fibrinogen concentrate with a target value of fibrinogen activity level of >1.5 g/L is recommended. In case of life-threatening hemorrhage, a target value of fibrinogen activity of >2 g/L should be used. Concerning antithrombotic therapy, the advice was to bridge fenprocoumon with LMWH in case of a surgical intervention. Anticoagulant treatment in patients with CFDs is in concordance with the recommendations for the general population (4, 20). Direct anticoagulants are the treatment of first choice, LMWH is the treatment of second choice. Vitamin K antagonists can be used as well, but can be difficult to monitor as a pre-existing PT prolongation can influence the INR, complicating the monitoring of the target INR value (4, 20). Our proband had a normal pre-existing INR and treatment with fenprocoumon was chosen because of recurrent PE under dabigatran treatment.

For both daughters, a similar treatment plan was set up concerning management in case of bleeding and dental and/or surgical interventions. Guiding advice during pregnancy and delivery was added. In case of pregnancy, it was recommended to start with a prophylactic dose of LMWH. During delivery, a target value of fibrinogen activity of >1.5 g/L is recommended. In case of post-partum hemorrhage, a target value of fibrinogen activity of >2.0 g/L is recommended, including treatment with fibrinogen concentrate. Taking into consideration the prothrombotic risk of oral contraceptives, the proband's daughters were advised to switch to another form of contraception, such as the use of an intrauterine device (IUD) (21, 22).

## 5 Discussion

The diagnostic workup of CFDs is relatively straightforward, but managing these patients is challenging as they can present with both bleeding and thrombotic manifestations. While a prolonged PT and low fibrinogen activity might indicate a bleeding tendency, conventional laboratory tests do not provide any indications for a thrombotic diathesis. This case report illustrates the utility of specialized research hemostasis tests, such as thrombin generation and thrombus formation under flow, in elucidating the clinical phenotype in three members of a hypodysfibrinogenemic family with a novel *FGG* mutation.

Thrombin generation at low TF showed slightly shorter lag times and higher peak heights in both daughters compared to normal pooled plasma, which was largely due to oral contraceptive use at the time of testing. Remarkably, however, the thrombin peak measured in the plasma of daughter 2 after discontinuation of the pill was still similar to that of normal plasma, whereas her fibrinogen antigen level (−1 mg/mL) would predict a lower peak height (8, 23, 24). Whether this discrepancy is attributable to qualitative properties of her mutant fibrinogen or to other determinants of thrombin generation in her plasma remains to be elucidated. Based on thrombin generation at high TF in the absence and presence of TM, both daughters also had APC-resistance, which persisted in daughter 2 after discontinuation of oral contraceptive use. This may be explained, at least in part, by her low fibrinogen level, as fibrinogen (and particularly fibrinogen γ') have been shown to counteract APC resistance (25). Overall, our findings seem in line with a previous study of thrombin generation performed with ST Genesia® in 22 patients with CFDs, where peak height and ETP were found to be inversely correlated with fibrinogen activity in both the STG-BLS (low TF) and STG-TS-TM (higher TF with TM) variants of the assay (6).

Microfluidic testing was performed to further investigate the overall hemostatic profile under shear. Two remarkable findings of these experiments were (i) a higher aggregate coverage on the collagen III spot in both daughters and (ii) fibrin formation on all three spots in both daughters, even in the absence of extrinsic triggering of coagulation. This is noteworthy, because in healthy controls, fibrin formation is only observed on the collagen I spot co-coated with TF. These results indicate a hypercoagulable rather than a hypocoagulable state in both daughters (without clinical manifestations to date). Fibrin formation on the collagen I spot and the collagen III spot is not expected in our flow model, since extrinsic activation with TF is needed to induce the coagulation cascade. The findings in both daughters are unlikely to be artefacts, as they were not observed in the controls, which were measured under the same circumstances. Moreover, repeated microfluidic testing in daughter 2, three months after stopping the use of oral contraceptives, showed similar results, indicating reproducibility over time despite discontinuation of oral contraceptives.

Unfortunately, thrombin generation and microfluidic assays are rarely available in clinical settings and their poor automatization and standardization still limits their clinical applicability. Another major drawback is that they do not probe fibrinolysis, abnormalities of which may contribute to both bleeding and thrombotic tendencies.

Besides its procoagulant function as a precursor of fibrin, fibrinogen has been reported to exert an anticoagulant activity known as “antithrombin I” (26). The main effector of this activity is the fibrinogen γ’ fraction, which has been shown to bind thrombin with high affinity and to inhibit its ability to activate platelets, FV and FVIII (15–17). The physiological relevance of these effects is supported by the association of the common *FGG* H2 haplotype and other genetic variants predicting relatively lower fibrinogen γ' levels with an increased risk of venous thrombosis (18, 24). Therefore, it is possible that the lower levels of fibrinogen (γ') in the family under study promotes a hypercoagulable state (26, 27). However, since not all hypo(dys)fibrinogenemias are associated with venous thrombosis, as was also observed in a recently published study on CFD's (28), it is likely that other qualitative properties of fibrinogen conferred by the *FGG* p.Tyr375Cys mutation also contribute to a prothrombotic state, possibly by altering cloth strength, structure or stability (27). Additional studies specifically focusing on clot formation, structure and degradation would be required to address this issue and to shed more light on the prothrombotic mechanisms of this particular fibrinogen variant.

## 6 Conclusion

In summary, this case report describes a family with three cases of hypodysfibrinogenemia, caused by a p.Tyr375Cys substitution in the fibrinogen gamma chain and characterized by a prothrombotic tendency. In vitro, a hypercoagulable state was exhibited. Hypodysfibrinogemia can induce both thrombotic and bleeding phenotypes, which emphasizes the importance of understanding the functional defects on the molecular level. In this case, the thrombin generation assay and particularly thrombus formation measurements using the Maastricht Flow Chamber detected a hypercoagulable state in both daughters and changed their treatment plan. The combination of quantitative and functional laboratory tests may have the potential to bridge the gap between the genotype and clinical phenotype in the future.

## Data Availability

The raw data supporting the conclusions of this article will be made available by the authors, without undue reservation.
