# Supplementary material for: Case Report: Laboratory detection of a thrombotic tendency in a family with hypodysfibrinogenemia and a novel FGG mutation
Source: Front Cardiovasc Med. 2024 Oct 15;11:1488602. doi: 10.3389/fcvm.2024.1488602 (PMC11518780; doi:10.3389/fcvm.2024.1488602)
Supplement: Supplementary file 1 [file Datasheet1.pdf]

Supplementary Material

Supplementary Figure S1:

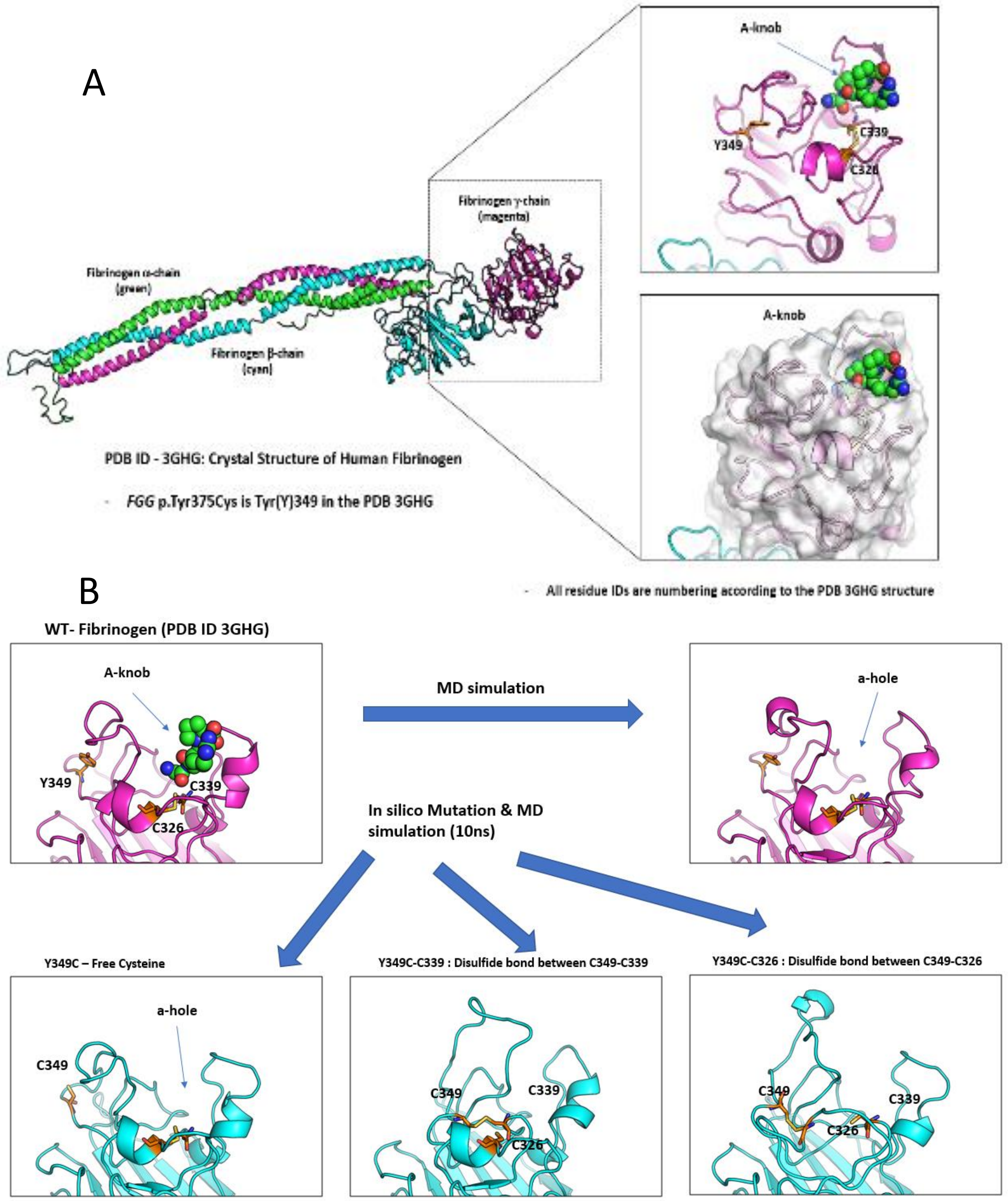

Figure S1. ***In silico*** **analysis of the** ***FGG*** **p.Tyr375Cys mutation.** A) Crystal structure of human fibrinogen (PDB 3GHG). The fibrinogen α-chain, β-chain, and γ-chain are shown in green, cyan and magenta, respectively. The residue affected by the mutation (*FGG* p.Tyr375Cys), which corresponds to Tyr(Y)349 in the PDB structure, is displayed in orange stick. The residue IDs are labelled according to the PDB structure (3GHG).

B) *In silico* mutation and molecular dynamics (MD) simulation of the wild-type fibrinogen (PDB ID 3GHG) (top) and the p.(Tyr375Cys) amino acid substitution (bottom). The p.(Tyr375Cys) mutation can lead to three possible scenarios: free cysteine at position C349 (bottom left), a disulfide bond

between C349 and C339 (bottom middle), and a disulfide bond between C349 and C326 (bottom right).

Supplementary Figure S2:

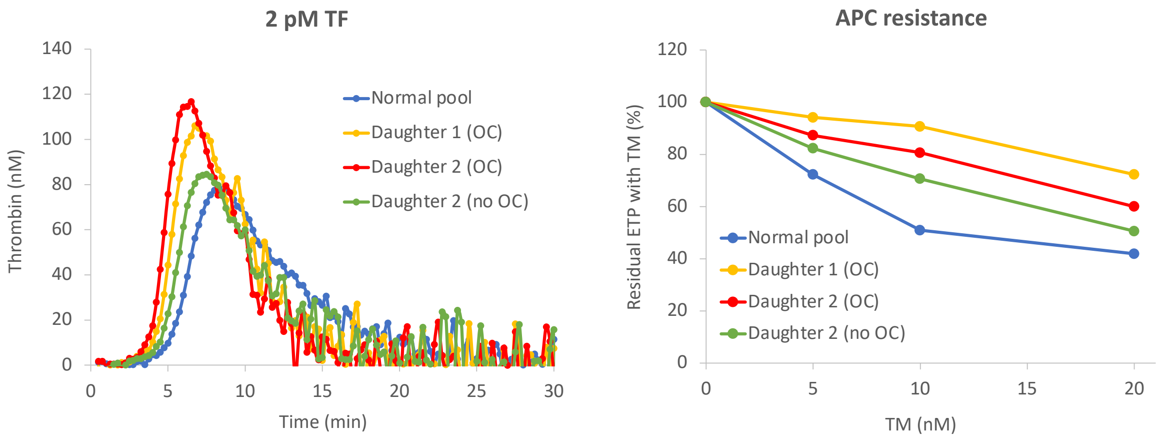

Figure S2. Left: Thrombin generation was measued in PPP using the CAT-method after initiation of coagulation with 2 pM tissue factor (TF). Right: Thrombin generation was measured at 10 pM TF in the presence of increasing concentrations (0-20 nM) of soluble thrombomodulin (sTM). The ratio between the endogenous thrombin potentials (ETP) determined in the presence and absence of TM (expressed in %) was taken as a measure of activated protein C (APC) resistance.
TF: tissue factor; APC: activated protein C; OC: oral contraceptives.

Supplementary Figure S3:

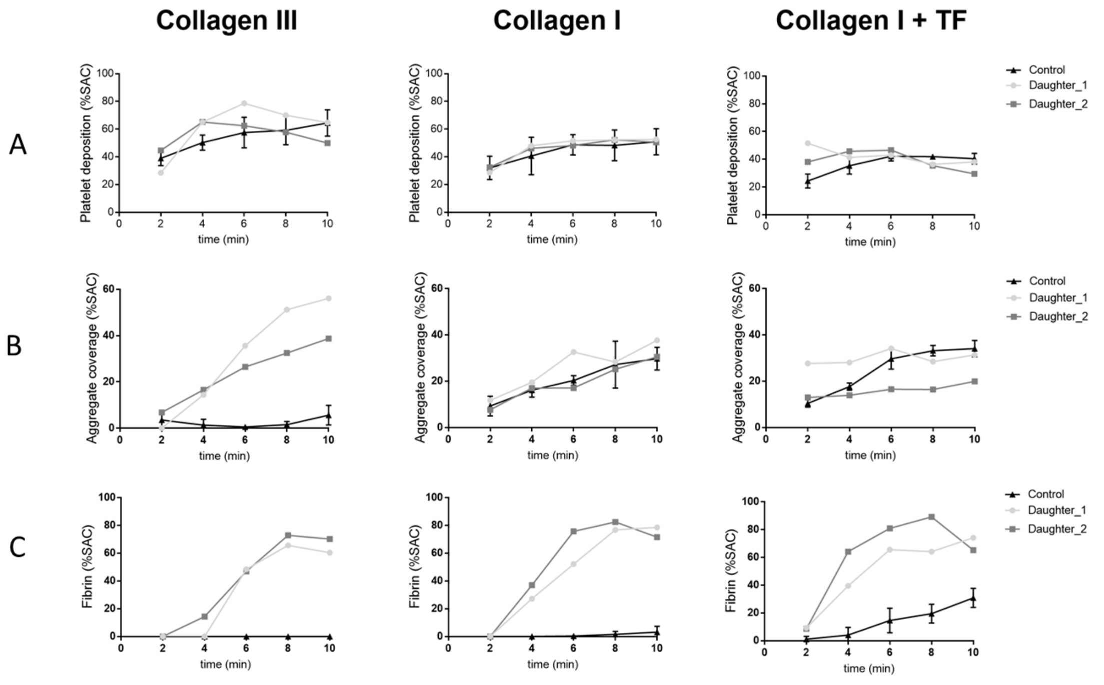

Figure S3. Platelet deposition, aggregate coverage and fibrin formation are quantified as percentage of surface area coverage (%SAC) and obtained from images taken at t=2, 4, 6, 8 and 10 minutes. Data obtained from daughters 1 and 2 were compared to 6 healthy donors. Control data are mean ± SD. Panel A: Platelet deposition in daughter 1 and 2 on oral contraceptive. Panel B: aggregate coverage in daughter 1 and 2 on oral contraceptive. Panel C: fibrin formation in daughter 1 and 2 on oral contraceptive.
